# Hec1/Ndc80 Tail Domain Function at the Kinetochore-Microtubule Interface

**DOI:** 10.3389/fcell.2020.00043

**Published:** 2020-02-26

**Authors:** Robert T. Wimbish, Jennifer G. DeLuca

**Affiliations:** Department of Biochemistry and Molecular Biology, Colorado State University, Fort Collins, CO, United States

**Keywords:** mitosis, kinetochore, microtubule, NDC80, Hec1

## Abstract

Successful mitotic cell division is critically dependent on the formation of correct attachments between chromosomes and spindle microtubules. Microtubule attachments are mediated by kinetochores, which are large proteinaceous structures assembled on centromeric chromatin of mitotic chromosomes. These attachments must be sufficiently stable to transduce force; however, the strength of these attachments are also tightly regulated to ensure timely, error-free progression through mitosis. The highly conserved, kinetochore-associated NDC80 complex is a core component of the kinetochore-microtubule attachment machinery in eukaryotic cells. A small, disordered region within the Hec1 subunit of the NDC80 complex – the N-terminal “tail” domain – has been actively investigated during the last decade due to its roles in generating and regulating kinetochore-microtubule attachments. In this review, we discuss the role of the NDC80 complex, and specifically the Hec1 tail domain, at the kinetochore-microtubule interface, and how recent studies provide a more unified view of Hec1 tail domain function.

## Introduction

**FIGURE 1:**
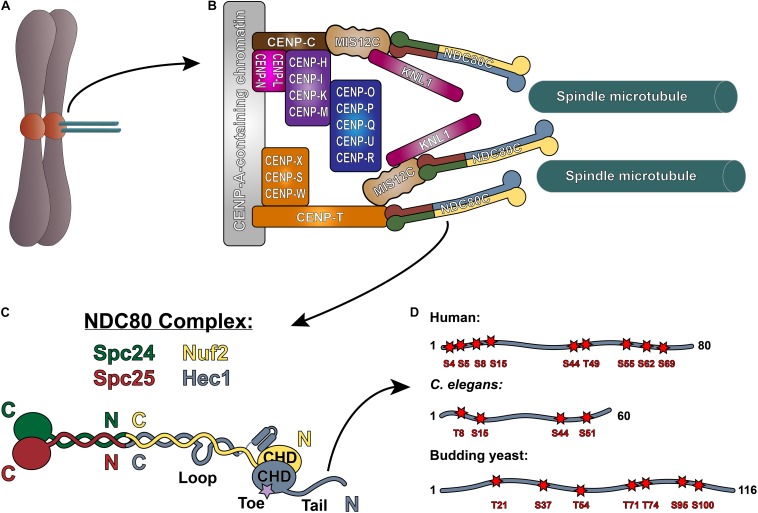
The NDC80 complex at the kinetochore-microtubule interface. **(A)** Mitotic chromosome. **(B)** Organization of the kinetochore-microtubule interface in vertebrate cells. The foundation of the kinetochore is the CCAN, or the Constitutive Centromere Associated Network, which binds to CENP-A-containing centromeric chromatin. The CCAN is composed of 16 subunits, organized in multiple subcomplexes including: CENP-L/N; CENP-O/P/Q/U/R; CENP-H/I/K/M; CENP-T/W/S/X; and CENP-C. CENP-C recruits the KMN “network” (composed of KNL1, the MIS12 complex, and the NDC80 complex) through its direct association with the MIS12 complex. CENP-T also recruits the NDC80 complex alone, as well as the KMN network through binding the MIS12 complex. **(C)** Architecture of the NDC80 complex. The C-termini of Spc24 (green) and Spc25 (red) form the kinetochore-targeting domain which binds either the MIS12 complex or CENP-T. The N-terminal regions of Spc24 and Spc25 form a coiled-coil domain that tetramerizes with the C-termini of Nuf2 (yellow) and Hec1 (blue). The N-terminus of Hec1 is comprised of a well-ordered CH domain, which contains the high affinity microtubule-binding “toe” region, and the tail domain which is also implicated in microtubule binding. The ∼40 amino acid loop domain of Hec1 is also indicated on the schematic. **(D)** Representation of the Hec1/Ndc80 tail domains from human, *Caenorhabditis elegans*, and the budding yeast *Saccharomyces cerevisiae*. Shown are the mapped and putative Aurora kinase phosphorylation sites. The human sites shown are Ser4, Ser5, Ser8, Ser15, Ser44, Thr49, Ser55, Ser62, and Ser69. The *C. elegans* sites shown are Thr8, Ser18, Ser44, and Ser51. The budding yeast sites shown are Thr21, Ser37, Thr54, Thr71, Thr74, Ser95, and Ser100 (see text for references).

**FIGURE 2:**
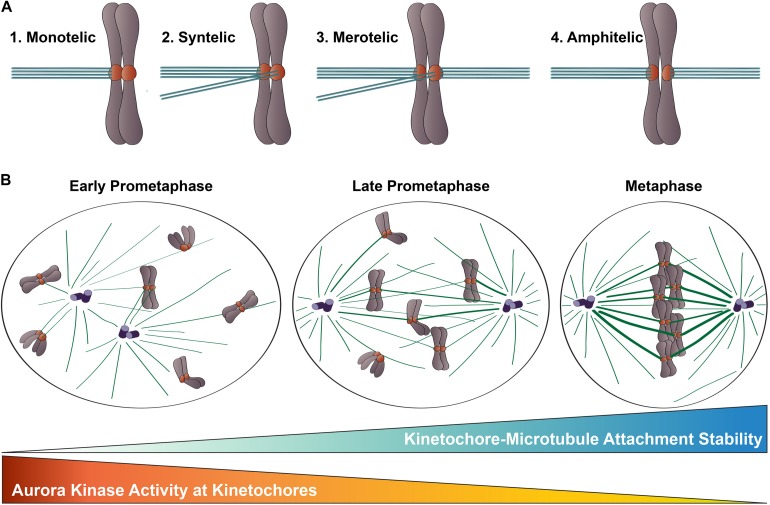
Kinetochore-microtubule attachments during mitosis. **(A)** Types of kinetochore-microtubule attachments. (1) Monotelic attachment: one sister kinetochore is attached to microtubules from one spindle pole and one sister is unattached; (2) Syntelic attachment: both sister kinetochores are attached to microtubules emanating from the same spindle pole; (3) Merotelic attachment: one sister kinetochore is attached to microtubules from both spindle poles; and (4) Amphitelic attachment (correct): one sister kinetochore is attached to microtubules from one pole and one sister kinetochore is attached to microtubules from the opposite pole. **(B)** Chromosome congression during mitotic progression. In early prometaphase, kinetochore-microtubule attachments errors are common, kinetochore-microtubule attachments are short-lived and labile, and Aurora B kinase activity at kinetochores is high. As mitosis progresses, erroneous kinetochore-microtubule attachments are corrected, kinetochore-microtubule attachments become long-lived and stable, and Aurora B kinase activity at kinetochores decreases.

Congression of mitotic chromosomes relies on interactions between spindle microtubules and kinetochores, which are comprised of a large number of proteins and multi-protein complexes assembled on regions of centromeric heterochromatin within each sister chromatid (Figures 1A,B). Kinetochores face the challenging task of directly binding to microtubule plus ends and tracking with them as they undergo cycles of polymerization and depolymerization. By doing so, kinetochores are able to harness the forces generated by microtubule dynamics to power chromosome movements that result in their alignment at the spindle equator. Equally importantly, kinetochores must regulate the strength with which they bind microtubules to ensure that incorrect attachments are released and correct attachments are stabilized, which is essential to prevent chromosome mis-segregation at mitotic exit (Figure 2).

At the core of the kinetochore’s force-transducing microtubule binding activity is the NDC80 complex, a heterotetrameric protein complex comprised of Hec1 (also called Ndc80), Nuf2, Spc24, and Spc25. Extending roughly 60 nm in length, the NDC80 complex is a dumbbell-shaped structure with two globular domains on each end, connected by a central coiled-coil shaft (Ciferri et al., 2005, 2008; Wei et al., 2005, 2006; Wang et al., 2008; Figure 1C). At one end of the complex, the C-terminal domains of Spc24 and Spc25 each adopt a RWD (RING finger, WD repeat, DEAD-like helicase) fold, through which they associate with either the Mis12 complex (bound to CENP-C or CENP-T) or CDK1-phosphorylated CENP-T to anchor the NDC80 complex to the kinetochore (Wei et al., 2006; Ciferri et al., 2008; Petrovic et al., 2010; Malvezzi et al., 2013; Nishino et al., 2013; Huis in’t Veld et al., 2016; Hara et al., 2018; Figure 1B). The N-terminal regions of Spc24 and Spc25 form a coiled-coil domain, which associates with the long coiled-coil domain of the Hec1/Nuf2 dimer at a tetramerization junction (Ciferri et al., 2005; Wei et al., 2005; Valverde et al., 2016). The Hec1/Nuf2 coiled-coil domain, which accounts for nearly 40 nm of the NDC80 complex’s length, is interrupted briefly by a ∼40 amino acid region in Hec1, termed the “loop” domain (Figure 1C; Maiolica et al., 2007). At the end of the NDC80 complex opposite the kinetochore-docking region, the N-termini of Hec1 and Nuf2 fold into a dimerized pair of globular calponin-homology (CH) domains (Wei et al., 2007; Ciferri et al., 2008), a conserved fold found in both actin and microtubule binding proteins (Slep et al., 2005; Sjöblom et al., 2008). The Hec1 CH domain contains a high-affinity microtubule binding site – termed the “toe” – that docks into the microtubule lattice between tubulin monomers at both the inter- and intra-dimer interfaces (Wilson-Kubalek et al., 2008; Alushin et al., 2010; Figure 1C). In all organisms tested to date, mutations in this region – even single point mutations – abolish kinetochore-microtubule interactions in cells and significantly weaken NDC80 complex-microtubule binding *in vitro* (Ciferri et al., 2008; Sundin et al., 2011; Tooley et al., 2011; Cheerambathur et al., 2013; Lampert et al., 2013). At its extreme N-terminus, Hec1 contains a positively charged, unstructured region that varies in length from ∼60–116 amino acids, depending on the organism (Figure 1D). A large body of work in cells and *in vitro* has demonstrated that this N-terminal region – termed the Hec1 “tail” domain – plays at least two distinct roles in kinetochore function: (1) phosphorylation of the tail by the Aurora family of kinases regulates kinetochore-microtubule attachment stability; and (2) the tail contributes to the establishment and maintenance of force-generating kinetochore-microtubule attachments in cells. It is unclear if these two functions of the Hec1 tail are conserved across species, as its contribution to these processes differ between organisms. In the following sections, we will discuss our current understanding of the role of the Hec1 tail domain in phosphoregulation of kinetochore-microtubule attachments, and in generating and maintaining force-transducing attachments during mitosis.

## Phosphoregulation of Kinetochore-Microtubule Attachments

During mitotic progression, a critical function of kinetochores is to adjust the strength with which they bind to spindle microtubules to control kinetochore-microtubule attachment stability. In early mitosis, kinetochore-microtubule attachments are unstable and undergo rapid turnover as a consequence, thus enabling improper attachments to be “reset” until correct, amphitelic attachments are established (Figure 2A). As mitosis progresses, the number of microtubules bound to each kinetochore increases, leading to stable attachments that can transduce the forces generated by microtubule dynamics to drive chromosome movement (Zhai et al., 1995; Salmon et al., 2005; Cimini et al., 2006; DeLuca et al., 2006; Bakhoum et al., 2009; Godek et al., 2015; Figure 2B). This increase in microtubule occupancy at each kinetochore also serves to silence the spindle assembly checkpoint, which is a quality-assurance mechanism cells use to prevent anaphase onset until all kinetochores are properly attached to spindle microtubules (Etemad et al., 2015; Krenn and Musacchio, 2015). Aurora B kinase, the enzymatic component of the Chromosomal Passenger Complex (CPC) that localizes to centromeres and kinetochores of mitotic chromosomes, is a member of the conserved Aurora family of serine/threonine protein kinases (Carmena et al., 2012; Hindriksen et al., 2017). This essential kinase has been recognized as the “master regulator” of kinetochore-microtubule attachment stability for almost 20 years, and a large body of work in multiple organismal systems has demonstrated that Aurora B kinase activity at kinetochores promotes turnover of kinetochore-attached microtubules, which in turn, prevents premature stabilization and accumulation of erroneous attachments during mitosis (Biggins et al., 1999; Kallio et al., 2002; Murata-Hori and Wang, 2002; Tanaka et al., 2002; Ditchfield et al., 2003; Hauf et al., 2003; Lampson et al., 2004; Figure 2). The Hec1 tail domain has since been identified as a key substrate of Aurora B kinase, and numerous studies from the last decade provide compelling evidence that phosphorylation of this domain serves as a major effector of Aurora B kinase’s regulation of kinetochore-microtubule attachment stability in metazoan cells (Cheeseman et al., 2006; DeLuca et al., 2006, 2011, 2018; Nousiainen et al., 2006; Kettenbach et al., 2011). Exactly how phosphorylation of the Hec1 tail domain carries out this important role has been a focus of research in recent years, and below we review the progress in addressing this question.

### Roles for Hec1 Tail Domain Phosphorylation at the Kinetochore-Microtubule Interface

#### Regulation of Kinetochore-Microtubule Attachment Stability

Initial evidence suggesting a role for the Hec1 tail domain in regulating kinetochore-microtubule attachment stability came from a study in PtK1 cells (derived from female rat kangaroo kidney epithelium), in which cells were microinjected with an antibody directed to the N-terminus of Hec1 (DeLuca et al., 2006). Injected cells formed hyper-stable kinetochore-microtubule attachments, as evidenced by (1) increased kinetochore-microtubule lifetimes and inter-kinetochore distances (stretched centromeres), (2) a high frequency of kinetochore-microtubule attachment errors which persisted into anaphase, and (3) dampened kinetochore oscillations. *In vitro* kinase assays and mass spectrometry analysis revealed that a recombinantly expressed Hec1^1–230^ fragment was phosphorylated by Aurora B kinase on multiple sites in its far N-terminal domain. Mutagenesis of these target sites to prevent phosphorylation partially recapitulated the microinjection results, suggesting that the injection phenotypes were, at least in part, due to loss of Hec1 tail domain phosphoregulation (DeLuca et al., 2006). These results were corroborated by subsequent studies in rat kangaroo, human, chicken, and *Caenorhabditis elegans* cells, in which Aurora B kinase target residues in the Hec1 tail were mutated to prevent phosphorylation (by alanine substitution) or to mimic phosphorylation (by substitution with either aspartic acid or glutamic acid). In these studies, preventing phosphorylation of the Hec1 tail resulted in hyper-stabilization of kinetochore-microtubule attachments, while mimicking phosphorylation led to unstable attachments in cells (Guimaraes et al., 2008; Welburn et al., 2010; DeLuca et al., 2011; Sundin et al., 2011; Cheerambathur et al., 2013; Zaytsev et al., 2014). Coincident with in-cell studies, *in vitro* microtubule binding experiments using recombinantly expressed, purified NDC80 complexes provided insight into the mechanism for this phosphoregulation. In the first of these, Cheeseman et al. (2006) demonstrated that purified *C. elegans* NDC80 complexes phosphorylated by Ipl1 (the budding yeast Aurora kinase) bound microtubules with significantly lower affinity than unphosphorylated complexes. Subsequent studies reported similar decreases in binding affinity for microtubules *in vitro* using purified human NDC80 complexes assembled with mutants of Hec1 containing phospho-mimetic substitutions at Aurora B kinase target sites (Alushin et al., 2012; Umbreit et al., 2012; Zaytsev et al., 2015). Together, these *in vitro* and cell-based studies substantiate a model in which phosphorylation of the Hec1 tail domain decreases the affinity of the NDC80 complex for microtubules, which consequentially decreases the attachment strength between kinetochores and spindle microtubules (Figure 2B).

It is noteworthy to mention that phosphoregulation of kinetochore-microtubule attachments differs somewhat in budding yeast compared to metazoans. While the budding yeast Hec1/Ndc80 tail domain contains multiple Aurora kinase phosphorylation consensus sites – at least four of which are phosphorylated in cells (Cheeseman et al., 2002; Akiyoshi et al., 2009) – neither mimicking nor preventing phosphorylation on these sites impacts cell viability or growth rates (Akiyoshi et al., 2009; Kemmler et al., 2009). Despite this, Ipl1 – the Aurora B kinase homolog in budding yeast – is required for cell viability (Francisco et al., 1994), suggesting that other Aurora B/Ipl1 targets distinct from the Hec1/Ndc80 tail domain are critical in budding yeast. The most likely candidate for this is the Dam1 complex, a 10-subunit kinetochore-associated complex that binds to both microtubules and the NDC80 complex, and which is not present in metazoans (Cheeseman et al., 2001; Jones et al., 2001; Janke et al., 2002; Shang et al., 2003; Miranda et al., 2005; Westermann et al., 2005; Lampert et al., 2010; Tien et al., 2010). Multiple subunits within the Dam1 complex are phosphorylated by Aurora/Ipl1 kinase, which leads to a reduced affinity of Dam1 for NDC80 complexes *in vitro* (Shang et al., 2003; Tien et al., 2010; Kim et al., 2017). Consistent with these data, cells expressing phospho-mimetic Dam1 mutants exhibit reduced kinetochore recruitment of Dam1 complexes and undergo defective chromosome segregation (Cheeseman et al., 2002; Shang et al., 2003; Kalantzaki et al., 2015; Jin et al., 2017), suggesting that Dam1 is a key target of Aurora/Ipl1 kinase in the regulation of kinetochore-microtubule attachment stability in budding yeast.

#### The Hec1 Tail Domain and Microtubule Dynamics

Umbreit et al. (2012) demonstrated that recombinant human NDC80 complexes, when linked to beads at relatively high density, were able to track dynamic microtubule ends *in vitro*, even when an external force was applied by an optical trap. This group also noted that the bead-bound NDC80 complexes promoted microtubule rescue events, in which microtubule ends switch from a state of depolymerization to one of polymerization. Rescue events were not observed with similarly bead-bound phospho-mimetic NDC80 mutant complexes (9D-Hec1, in which all nine Aurora kinase target sites in the tail domain are mutated to aspartic acid) (Umbreit et al., 2012). This inability to promote rescue events was not due to the fact that 9D-Hec1-containing NDC80 complexes bound more weakly to microtubules, because complexes lacking the entire Hec1 tail, which bound to microtubules as poorly as those containing 9D-Hec1, were capable of promoting some degree of rescue (Umbreit et al., 2012). These results bring to light the interesting possibility that phosphorylation of the Hec1 tail may not only promote release of kinetochore-bound microtubules, but may also promote plus-end microtubule depolymerization. This idea is consistent with an earlier study demonstrating that syntelically attached sister kinetochore pairs initiate Aurora B kinase-mediated error correction with rapid poleward movement along depolymerizing microtubules (Lampson et al., 2004). A role for Hec1 tail phosphorylation in kinetochore-mediated regulation of microtubule dynamics could also help explain the well-documented phenotype of dampened kinetochore oscillations in cells expressing non-phosphorylatable 9A-Hec1 mutants (DeLuca et al., 2011; Zaytsev et al., 2014; Long et al., 2017). Reduced kinetochore oscillatory behavior is typically attributed to hyper-stable kinetochore-microtubule attachments, which lead to increased frictional forces that ultimately reduce kinetochore mobility (DeLuca et al., 2011; Zaytsev et al., 2014; Long et al., 2017). It is also plausible, however, that preventing phosphorylation of the Hec1 tail domain leads to increased rescue frequency, and thus decreased dynamics of the kinetochore-bound microtubules, which could result in dampened oscillations. Determining if the dynamic behavior of microtubule ends can be “tuned” *in vitro* by the phosphorylation state of the Hec1 tail domain would shed light on this interesting question.

In-cell and *in vitro* assays have allowed investigation into how phosphorylation of the Hec1 tail affects the ability of NDC80 complexes to track with and transduce forces from polymerizing and depolymerizing microtubules. In a recent study, Long et al. (2017) used laser ablation in PtK1 cells to sever metaphase kinetochore fibers and differentially induce sister kinetochores to move either poleward, along mostly depolymerizing microtubules, or anti-poleward, along mostly polymerizing microtubules, in order to investigate how Hec1 tail phosphorylation affects the tracking behavior of kinetochores. By quantitating kinetochore movements after laser ablation, the authors found that preventing Hec1 tail phosphorylation significantly decreased the velocity of sister kinetochores moving anti-poleward, while the velocity of those moving poleward was unaffected (Long et al., 2017). This led the authors to conclude that phosphorylation of the Hec1 tail regulates the kinetochore’s affinity for polymerizing, but not depolymerizing microtubules. *In vitro*, NDC80 complexes bind more weakly to depolymerizing microtubule ends than to polymerizing ends, and this has been attributed to a lower affinity of the Hec1 CH domain for curved microtubule protofilaments (which are formed at microtubule ends during depolymerization, McIntosh et al., 2008) than for straight protofilaments (Alushin et al., 2010; Schmidt et al., 2013). Thus, it is possible that in cells, the majority of kinetochore-bound NDC80 complexes unbind from depolymerizing ends, and attachments are maintained by other kinetochore-associated microtubule binding proteins. Unbinding of NDC80 complexes from microtubules may explain why Long et al. (2017) found that the phosphorylation state of the tail domain does not impact velocities of kinetochores moving poleward. Recent experiments from Yoo et al. (2018), however, may argue against this idea. In their study, the authors employed FRET sensors (in tubulin and the Nuf2 subunit of the NDC80 complex) to measure the fraction of microtubule-bound NDC80 complexes during metaphase chromosome oscillations in human cells. While the authors reported a statistically significant decrease of microtubule-bound NDC80 complexes on poleward moving kinetochores (containing mostly depolymerizing microtubules) in comparison to those on anti-poleward moving kinetochores (containing mostly polymerizing microtubules), this difference was small (∼11% change in NDC80 complex FRET fraction), especially compared to the FRET change measured in early prometaphase with respect to late metaphase (∼50% change in FRET fraction; Yoo et al., 2018). These observations suggest that NDC80 complexes remain closely associated with the microtubule lattice on both the poleward and anti-poleward moving kinetochores of a sister pair. Furthermore, a recent study by Huis in’t Veld et al. (2019) investigated how the phosphorylation state of the Hec1 tail domain impacted the ability of human NDC80 complexes to maintain attachments to depolymerizing microtubules *in vitro*. The authors reported that while the phosphorylation state of the tail did not affect the ability of trimerized, bead-bound NDC80 complexes to track with depolymerizing microtubules in the absence of tension, when a resisting force was applied with an optical trap, phosphorylated NDC80 complexes detached from depolymerizing microtubules with significantly higher frequency than non-phosphorylated complexes (Huis in’t Veld et al., 2019). These results suggest that, at least *in vitro*, Hec1 tail phosphorylation affects the ability of human NDC80 complexes under tension to transduce forces from depolymerizing microtubules. Why the phosphorylation state of the tail domain affects kinetochore movement along anti-poleward moving, but not poleward-moving kinetochores in cells remains an important unanswered question.

### Temporal Regulation of Hec1 Tail Domain Phosphorylation

A key aspect of kinetochore-microtubule attachment regulation is the ability of kinetochores to impart changes in attachment stability from early to late mitosis: attachments are labile in early mitosis to prevent accumulation of erroneous connections, and stable in late mitosis to drive chromosome congression and mitotic exit (Zhai et al., 1995; Cimini et al., 2006; DeLuca et al., 2006; Bakhoum et al., 2009; Figure 2B). Cells primarily achieve this temporal regulation by modulating Hec1 phosphorylation levels. In 2011, phospho-specific antibodies were generated against four Aurora B kinase target sites in the Hec1 tail (serines 8, 15, 44, and 55), and were used to monitor phosphorylation levels at kinetochores during mitosis. All four sites were found to be phosphorylated at high levels in early prometaphase, and at much lower levels as cells progressed through metaphase and anaphase (DeLuca et al., 2011). Later studies found that expression of Hec1 mutants with increasing numbers of phospho-mimetic substitutions in the tail domain caused a corresponding decrease in kinetochore-microtubule attachment stability in cells (Zaytsev et al., 2014; Yoo et al., 2018; Etemad et al., 2019; Kuhn and Dumont, 2019). These findings were corroborated by *in vitro* data revealing a direct correlation between increasing number of phospho-mimetic substitutions in the Hec1 tail domain in purified human NDC80 complexes and decreasing microtubule binding affinity (Zaytsev et al., 2015). Together, these studies support a model in which phosphorylation of the Hec1 tail “tunes” kinetochore-microtubule attachment stability in cells by modulating the binding properties of NDC80 complexes for microtubules (Figure 2B).

An important question is how Aurora B kinase activity itself is regulated to effectuate differential phosphorylation of the Hec1 tail. The prevailing model that accounts for this regulation – the “spatial positioning” model – posits that Aurora B is recruited to the inner centromere in early mitosis from where it phosphorylates centromere proximal kinetochore substrates. Upon establishment of stable microtubule attachments, the outer kinetochore exhibits a tension-dependent stretch away from the centromere, thereby repositioning Aurora B substrates (e.g., Hec1 and other outer kinetochore targets) out of the kinase’s “reach,” and consequently reducing the extent of their phosphorylation (Biggins et al., 1999; Tanaka et al., 2002; Liu et al., 2009; Lampson and Cheeseman, 2011). A number of studies, however, have challenged this model. In chicken DT40 cells, preventing centromere targeting of Aurora B by mis-localizing the CPC component Survivin does not perturb chromosome segregation or cell viability (Yue et al., 2008). In human cells, Aurora B kinase is detected at the kinetochore itself in early mitosis (Posch et al., 2010; DeLuca et al., 2011), and mis-localization of Aurora B kinase away from kinetochores – but not from centromeres – leads to decreased Hec1 tail phosphorylation, and impaired kinetochore-microtubule error correction (Caldas et al., 2013; Hengeveld et al., 2017). Moreover, ectopic targeting of Aurora B kinase to centromeres does not restore phosphorylation of the Hec1 tail domain in cells that do not possess kinetochore-localized Aurora B (Caldas et al., 2013). Finally, in budding yeast, a mutation in the CPC component INCENP (Sli15 in *S. cerevisiae*) that disrupts its centromere localization, but not its microtubule binding activity, has no negative consequence on chromosome bi-orientation or Aurora B-mediated kinetochore-microtubule error correction (Campbell and Desai, 2013). Collectively, these studies suggest that Aurora B phosphorylation of kinetochore substrates is uncoupled from centromere accumulation of the kinase. An alternative “direct kinetochore binding” model posits that Aurora B kinase is recruited directly to unattached or incorrectly attached kinetochores where it phosphorylates Hec1 and other kinetochore substrates, and is subsequently evicted as kinetochores form stable, end-on attachments to microtubules (Caldas et al., 2013; Krenn and Musacchio, 2015). Identifying the receptor(s) for Aurora B kinase (and the CPC) at the kinetochore is an important goal that will move the field toward a better understanding of how kinetochore-microtubule attachments are regulated. In budding yeast, significant progress has been made on this front, with the recent identification of the inner kinetochore COMA complex as a direct recruitment site for Aurora B kinase and the CPC (Fischböck-Halwachs et al., 2019; García-Rodríguez et al., 2019). It is not yet known if the human homolog of the COMA complex, the inner kinetochore-associated CENP-O/P/Q/U complex, plays an analogous role.

### Contribution of Aurora A Kinase to Hec1 Tail Domain Phosphorylation

The Aurora family of kinases in higher eukaryotes is comprised of three unique members: Auroras A, B, and C. While Aurora C functions primarily in meiotic cell division, Auroras A and B both have essential and distinct functions in mitosis (reviewed in Barr and Gergely, 2007; Vader and Lens, 2008; Krenn and Musacchio, 2015; DeLuca, 2017). Aurora A and B kinases share a high degree of sequence similarity and recognize nearly identical consensus sequences in target substrates (Meraldi et al., 2004; Ohashi et al., 2006; Carmena et al., 2009; Kim et al., 2010; Kettenbach et al., 2011; Koch et al., 2011). Substrate specificity is thought to be attained in cells through differential sub-cellular localization: while Aurora B localizes to and phosphorylates substrates at centromeres and kinetochores, Aurora A localizes to and phosphorylates spindle pole-localized substrates (reviewed in Vader and Lens, 2008; Carmena et al., 2009; DeLuca, 2017). In a recent development, the Hec1 tail was identified as a bona fide Aurora A substrate in cells. Two studies published in 2015 – one carried out in mitotic and one in meiotic cells – reported that spindle pole-associated Aurora A phosphorylates Hec1 in early mitosis at serine 55 (Chmátal et al., 2015; Ye et al., 2015), a site previously demonstrated to be phosphorylated by Aurora B kinase in cells (DeLuca et al., 2011). In the mitotic study, overexpression of Aurora A in *Drosophila* S2 cells led to a reduced incidence of experimentally induced syntelic attachments, and depletion of Aurora A from PtK1 cells resulted in a significant increase in spindle pole-associated chromosomes (Ye et al., 2015). The authors of the meiotic study found that kinetochore-microtubule attachments of chromosomes in close proximity to one of the two spindle poles were destabilized in an Aurora A kinase-dependent manner (Chmátal et al., 2015). These studies, together with the finding that Aurora B kinase is enriched on kinetochores of spindle pole-proximal chromosomes (Maia et al., 2010; DeLuca et al., 2011), suggest that syntellically attached kinetochores “trapped” near a spindle pole likely employ both Aurora A- and B-mediated Hec1 tail domain phosphorylation for microtubule release and error correction. More recently, Hec1 serine 69 was identified as a physiologically important target of Aurora A kinase in human cells (DeLuca et al., 2018). In this study, inhibition of Aurora A, but not Aurora B kinase activity, resulted in a near-complete loss of serine 69 phosphorylation at kinetochores. Strikingly, phosphorylation of serine 69 persisted at high levels throughout the entirety of mitosis, unlike the other Hec1 tail domain target sites (DeLuca et al., 2018). Specifically preventing Aurora A-mediated phosphorylation of serine 69 resulted in significant dampening of metaphase kinetochore oscillations, suggesting that at least some level of sustained phosphorylation on the Hec1 tail is required for normal chromosome movements in late mitosis. Even more surprising, Aurora A kinase was detected at the kinetochores of mitotic chromosomes, and this localization was enhanced upon overexpression of the CPC component and Aurora B co-factor INCENP (DeLuca et al., 2018). Thus, in addition to functioning at spindle poles, Aurora A kinase plays an important role at kinetochores to regulate kinetochore–microtubule dynamics. How Aurora A kinase is recruited to kinetochores and how this recruitment is temporally regulated during mitosis remain important unanswered questions.

### Contribution of Mps1 Kinase to Hec1/Ndc80 Tail Domain Phosphorylation

In addition to containing Aurora B kinase consensus sites, the Hec1/Ndc80 tail domain also contains multiple consensus sites for the highly conserved spindle assembly checkpoint kinase Monopolar spindle 1 (Mps1) (Kemmler et al., 2009). While Mps1-mediated phosphorylation of the Hec1/Ndc80 tail domain remains to be investigated in mammalian cells, this domain is a bona fide Mps1 substrate in *S. cerevisiae* (Kemmler et al., 2009). Studies in budding yeast demonstrated that phospho-mimetic substitutions at 14 Mps1 target sites led to a failure of cells to silence the spindle assembly checkpoint, but had no effect on the establishment of kinetochore-microtubule attachments in cells, or on NDC80 complex-microtubule binding *in vitro* (Kemmler et al., 2009). The exact role for Mps1 phosphorylation of the tail domain in spindle assembly checkpoint signaling, and whether this mechanism is conserved in metazoan cells remains to be investigated.

### Hec1 Tail Domain Dephosphorylation

In contrast to the abundance of information available regarding the kinases that phosphorylate the Hec1 tail, there are surprisingly little data regarding the phosphatases that reverse these phosphorylation events. Studies from the last decade have suggested that dephosphorylation of kinetochore substrates during mitosis is facilitated largely by two phosphatases: PP1 and PP2A-B56 (reviewed in De Wulf et al., 2009; Funabiki and Wynne, 2013; Manic et al., 2017; Saurin, 2018). The activities of these two phosphatases reverse a large number of phosphorylation events at kinetochores, including those mediated by the Aurora kinase family. These dephosphorylation events have been implicated in stabilizing kinetochore-microtubule attachments and in silencing the spindle assembly checkpoint. Given the role of Hec1 tail domain phosphorylation in regulation of attachment stability, it follows that dephosphorylation plays an equally important role. PP1 localizes to kinetochores in late prometaphase and metaphase, as attachments become progressively stabilized (Trinkle-Mulcahy et al., 2003; Liu et al., 2010; DeLuca et al., 2011; Sivakumar et al., 2016), suggesting that Hec1 might be dephosphorylated by this phosphatase at these late stages of mitosis (Liu et al., 2010; DeLuca et al., 2011; Sivakumar et al., 2016). Several studies have demonstrated that preventing PP1 recruitment to kinetochores leads to major defects in spindle assembly checkpoint silencing (Pinsky et al., 2009; Vanoosthuyse and Hardwick, 2009; Meadows et al., 2011; Rosenberg et al., 2011; Espeut et al., 2012; London et al., 2012; Nijenhuis et al., 2014; Sivakumar et al., 2016; Kim and Stumpff, 2018), but the role of PP1 in stabilizing kinetochore-microtubule attachments is less clear. In *C. elegans*, preventing PP1 recruitment to its docking site on the kinetochore protein KNL1 delayed formation of load-bearing kinetochore-microtubule attachments; however, cells exhibited no obvious defects in chromosome segregation (Espeut et al., 2012). Although one study in human cells reported a similar finding – that preventing PP1 recruitment to KNL1 compromises kinetochore-microtubule attachments (Liu et al., 2010) – two recent studies found that kinetochore-microtubule attachments and chromosome alignment were both largely unperturbed upon disruption of the PP1-KNL1 interaction (Shrestha et al., 2017; Smith et al., 2019). Consistent with these latter results, Smith et al. (2019) reported no change in phosphorylation of Hec1 (on serine 55) at kinetochores in cells in which PP1-KNL1 recruitment was perturbed. These results suggest that dephosphorylation of the Hec1 tail domain by PP1 may not be a major effector of kinetochore-microtubule attachment regulation.

The other major phosphatase implicated in regulating kinetochore-microtubule attachment stability is PP2A-B56, which is recruited to kinetochores by the spindle assembly checkpoint effector BubR1 (Suijkerbuijk et al., 2012; Kruse et al., 2013; Xu et al., 2013). In contrast to PP1, inhibiting PP2A-B56 kinetochore recruitment leads to severe defects in chromosome alignment and kinetochore-microtubule attachments (Suijkerbuijk et al., 2012; Kruse et al., 2013; Xu et al., 2013; Shrestha et al., 2017; Smith et al., 2019). Consistent with these findings, cells expressing a mutant version of BubR1 that is unable to recruit PP2A-B56 to kinetochores exhibit increased levels of phosphorylated Hec1 (at serine 55) (Smith et al., 2019). Interestingly, the authors of this study found that they could rescue PP1 and PP2A-B56 depletion phenotypes by artificially recruiting the other phosphatase to kinetochores, suggesting that the phosphatases may have little intrinsic site specificity (Smith et al., 2019). PP1 and PP2A-B56 are recruited to kinetochores at different times during mitosis – PP2A-B56 in early mitosis, and PP1 in late mitosis – therefore, it is possible that the timing of their recruitment underlies their substrate specificity. It is worth noting that although most of the Aurora phosphorylation sites in the Hec1 tail domain are dephosphorylated by metaphase, serine 69 remains highly phosphorylated throughout the duration of mitosis (DeLuca et al., 2018). Given the proximity of the Hec1 tail domain phosphorylation target sites to each other, the difference in metaphase phosphorylation of serine 69 compared to the other target sites suggests there may in fact be some degree of phosphatase specificity. Determining which phosphatases are responsible for dephosphorylation of the individual sites in the tail domain will be important in resolving this issue.

### Hec1 Tail Domain Acetylation

While phosphorylation of the Hec1 tail is acknowledged as a critical mechanism for regulating kinetochore-microtubule attachment stability, it is important to note that other tail domain post-translational modifications may also contribute. Recent work has demonstrated that in human cells, the Hec1 tail is acetylated at lysines 53 and 59 by the acetyltransferase TIP60 (Zhao et al., 2019). In this study, the authors demonstrated that this modification exhibits crosstalk with the Aurora kinase pathway: notably, acetylation at these sites resulted in decreased Aurora-dependent phosphorylation at serines 55 and 62 *in vitro*, and preventing acetylation (through lysine to arginine mutation) destabilized kinetochore-microtubule attachments in cells (Zhao et al., 2019). These results raise the interesting possibility that kinetochore kinases and phosphatases need to coordinate not only with each other but with lysine acetylases and deacetylases to ensure proper regulation of kinetochore-microtubule attachment stability.

## Generation of Kinetochore-Microtubule Attachments

In addition to its role in regulating kinetochore-microtubule attachment stability, the Hec1 tail domain is also implicated in generating and maintaining stable, force-transducing kinetochore-microtubule attachments, but how it does so remains unclear. In the second part of this review, we will examine the functions of the Hec1 tail in forming these attachments in mitosis, and discuss how contributions of the tail to this process may vary among species.

### Hec1 Tail Contribution to NDC80 Complex-Microtubule Binding *in vitro*

Using a variety of *in vitro* approaches, multiple studies have demonstrated that recombinant NDC80 complexes and Hec1-Nuf2 dimers exhibit reduced binding affinity for microtubules in the absence of the Hec1 tail domain. Notably, this has been reported for NDC80 complexes (or complex components) from all species tested to date. For example, Wei et al. (2007) found a 7–10X reduction in microtubule binding affinity for the CH domains of the budding yeast NDC80 complex components Hec1/Ndc80 and Nuf2 when the N-terminal 116 amino acid tail domain was deleted. Ciferri et al. (2008) characterized the binding affinity of a tail deletion mutant of an engineered version of the human tetrameric NDC80 complex (lacking the majority of the internal coiled-coil region, termed NDC80^Bonsai^) and demonstrated that tail-less complexes exhibited decreased co-sedimentation with microtubules, with calculated binding affinities of ∼100X lower than wild-type complexes. These results reported for human NDC80^Bonsai^ complexes were later corroborated by Umbreit et al. (2012) using a TIRF-based fluorescence assay to characterize recombinantly expressed, full-length, GFP-tagged human NDC80 complexes. In population studies, NDC80 complexes lacking the Hec1 tail domain bound microtubules with ∼9X decreased affinity, and in single molecule studies, tail-less complexes exhibited an ∼14X increase in their dissociation rate from microtubules (Umbreit et al., 2012). A similar role for the tail was found using recombinant *C. elegans* NDC80 complexes, in which mutants lacking the N-terminal 60 amino acid Hec1/Ndc80 tail domain exhibited severely reduced microtubule binding affinity (Cheerambathur et al., 2013). Thus, the role of the tail domain in affecting the microtubule binding activity of the NDC80 complex appears to be conserved across species.

A recent study using engineered scaffolds to multimerize human NDC80 complexes has provided insight into how the tail domain might influence NDC80 complex-microtubule binding (Huis in’t Veld et al., 2019). The authors of this study found that bead-bound NDC80 complexes lacking the Hec1 tail exhibited almost wild-type microtubule residence times, in situations in which the tail-less NDC80 complexes were oligomerized on the bead surface (Huis in’t Veld et al., 2019). However, unlike wild-type complexes, these oligomerized tail-less NDC80 complexes were unable to track depolymerizing microtubule plus-ends. This effect may be due to the previously mentioned phenomenon that the NDC80 complex binds more weakly to curved, depolymerizing microtubule ends compared to straight, polymerizing ends (Alushin et al., 2010; Schmidt et al., 2013). Thus, the decreased microtubule binding affinity resulting from deletion of the Hec1 tail is likely compensated for by complex oligomerization on stabilized or polymerizing microtubules, but not on depolymerizing microtubules.

### Hec1 Tail Contribution to Kinetochore-Microtubule Attachments in Cells

Although it is well-established that the Hec1 tail domain contributes to high affinity microtubule binding *in vitro*, its role in forming stable kinetochore-microtubule attachments in cells is less clear. Budding yeast cells expressing Hec1/Ndc80 tail domain deletion mutants are viable, undergo normal chromosome segregation, and generate normal kinetochore-microtubule attachments (Kemmler et al., 2009; Demirel et al., 2012; Lampert et al., 2013). However, findings from a recent study indicate that the tail domain plays at least some role at the kinetochore-microtubule interface in this organism (Suzuki et al., 2016). By inserting a FRET-based sensor between the loop and CH domains of Hec1/Ndc80, the authors found that expression of the tail-less mutant resulted in decreased tension at the kinetochore-microtubule interface (Suzuki et al., 2016). They also noted that cells expressing the tail-less mutant experienced a prometaphase-to-anaphase delay, which led to a ∼10% increase in mitotic index. Thus, while the tail domain is not explicitly required for kinetochore-microtubule attachment in budding yeast, it has a role in force production at the attachment interface.

Consistent with observations in budding yeast, the Hec1/Ndc80 tail is not required for normal mitotic progression in *C. elegans*. Specifically, Cheerambathur et al. (2013) found that the kinetics of spindle pole separation in the first division of *C. elegans* embryos were unchanged in cells expressing Hec1/Ndc80 tail deletion mutants compared to wild-type embryos, which is indicative of normal kinetochore-microtubule attachments. Interestingly, the authors reported that the tail was required for interaction between the NDC80 complex and the RZZ complex component ROD-1. RZZ binding to the Hec1/Ndc80 tail was shown to negatively regulate kinetochore-microtubule attachments by inhibiting NDC80 complex-microtubule binding. The authors propose this mechanism is important in early mitosis to prevent premature stabilization of kinetochore-microtubule attachments. Whether this function is conserved in other eukaryotic systems remains an open question. Collectively, studies from budding yeast and *C. elegans* suggest that although the tail domain plays some role at the kinetochore-microtubule interface, it is not strictly required for productive attachments in cells.

In mammalian cells, the role of the Hec1 tail in generating stable kinetochore-microtubule attachments is not entirely resolved. Two studies published in 2008 reported that PtK1 and HeLa cells expressing Hec1 tail deletion mutants exhibited defects in chromosome alignment and mitotic progression, and failed to accumulate stable kinetochore-microtubule attachments (Guimaraes et al., 2008; Miller et al., 2008). More recent studies corroborated these findings by showing that expression of tail deletion mutants in HeLa cells leads to mitotic arrest and decreased inter-kinetochore distances (Etemad et al., 2015; Janczyk et al., 2017). In light of its requirement for high affinity NDC80 complex-microtubule interactions *in vitro*, these data led to the emergent view that the Hec1 tail domain is required for kinetochore-microtubule attachments in mammalian cells. However, recent work from our lab may challenge this notion. In particular, Wimbish et al. (2019) demonstrated that the tail is dispensable for the establishment and maintenance of kinetochore-microtubule attachments in HeLa and RPE1 cells. However, in spite of the presence of cold-resistant kinetochore-microtubule attachments, cells expressing a tail-less Hec1 mutant exhibited defects in chromosome alignment, and experienced long mitotic delays. Interestingly, stable kinetochore-microtubule attachments formed earlier in these cells compared with cells expressing wild-type Hec1. However, consistent with a role for the tail domain in contributing to force generation at the kinetochore-microtubule interface, inter-kinetochore distances were decreased between sister kinetochores of bi-oriented chromosomes, and cells failed to silence the spindle assembly checkpoint (Wimbish et al., 2019). Based on these phenotypes, we hypothesize that attachments are established prematurely in early mitosis due to an inability to negatively regulate attachments by Aurora kinase-mediated phosphorylation; however, in later mitosis, the tail is required to provide an additional contact point to microtubules to achieve wild-type levels of force generation. These recent results suggest that Hec1 tail domain functions at the kinetochore-microtubule interface may be more conserved among species than previously appreciated.

### Compensation for Hec1 Tail Function by Co-factors

Given the conserved role of the Hec1 tail domain in high affinity binding of NDC80 complexes to microtubules *in vitro*, an obvious question is why this domain is not ubiquitously required for stable kinetochore-microtubule attachments in cells. One likely explanation for this discrepancy is the presence of compensatory cellular factors that are missing from *in vitro* reconstitution experiments. In the case of budding yeast, this factor is likely the Dam1 complex. As noted previously, yeast cells expressing tail-less Hec1/Ndc80 mutants are viable; however, simultaneous expression of Hec1/Ndc80 tail deletion mutants and loss-of-function Dam1 mutants renders cells inviable (Demirel et al., 2012; Lampert et al., 2013). Consistently, Suzuki et al. (2016) demonstrated that budding yeast cells expressing tail-less Hec1/Ndc80 mutants and wild-type Dam1 exhibit decreased force generation at the kinetochore-microtubule interface; however, in spite of this, wild-type inter-kinetochore distances were maintained, indicating the presence of stable kinetochore-microtubule attachments. This led the authors to conclude that the Dam1 complex is able to compensate for loss of the Hec1/Ndc80 tail, and becomes the primary load-bearing complex at kinetochores in the absence of this domain (Suzuki et al., 2016). These findings are consistent with results from *in vitro* studies in which the microtubule binding activity of tail-less budding yeast NDC80 complexes is enhanced by the addition of Dam1 complexes (Lampert et al., 2010, 2013).

It has been appreciated for several years that the human Ska complex is able to increase NDC80 complex-microtubule binding affinity, and to enable end-tracking of NDC80 complexes on depolymerizing microtubules *in vitro* (Schmidt et al., 2013). Recent studies have suggested that, like the yeast Dam1 complex, Ska complexes can compensate for loss of the Hec1/Ndc80 tail domain in NDC80 complex-microtubule interaction assays. For instance, Helgeson et al. (2018) used optical trapping assays to show that Ska complexes can impart almost wild-type end-tracking activity to tail-less NDC80 complex-coated beads on depolymerizing microtubules, even under applied force. Similarly, Huis in’t Veld et al. (2019) demonstrated that Ska complexes can restore end-tracking activity to oligomerized tail-less complexes in the absence or presence of applied force. These results raise the possibility that the Ska complex may be able to, at least in part, compensate for the Hec1 tail domain in human cells. Indeed, a recent study from our lab demonstrated that HeLa cells depleted of Ska complex components and expressing a tail-less Hec1 mutant failed to establish stable kinetochore-microtubule attachments; however, this deficit could be restored when the Ska complex was left unperturbed (Wimbish et al., 2019). Thus, in a manner analogous to the coordinated activities of the NDC80 and Dam1 complexes in budding yeast (Kemmler et al., 2009; Demirel et al., 2012; Suzuki et al., 2016), the Ska complex likely functions in concert with the NDC80 complex in human cells in the establishment and maintenance of stable kinetochore-microtubule attachments.

## Mechanistic Perspectives of Hec1 Tail-Mediated Attachment Stabilization and Regulation

There is compelling evidence that the Hec1 tail domain plays a central role in the regulation of kinetochore-microtubule attachment stability and in the generation of force-transducing kinetochore-microtubule attachments during mitosis. Below we discuss three models, which are not mutually exclusive, that may explain how the Hec1 tail domain contributes to these critical mitotic functions.

### Models for Attachment Stabilization and Regulation by Direct Tail Domain-Microtubule Binding

**FIGURE 3:**
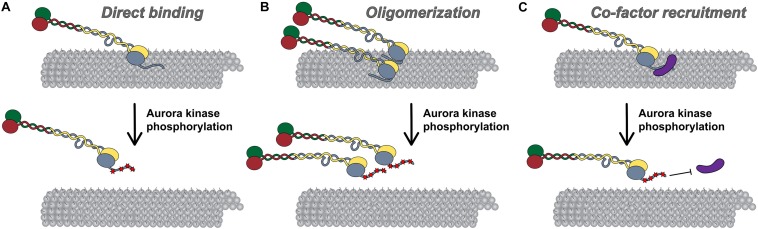
Models for Hec1 tail domain function. **(A)** Direct microtubule binding. In this model, the tail domain directly interacts with the microtubule lattice to increase CH-domain-mediated NDC80 complex-microtubule interactions. Phosphorylation of the Hec1 tail reduces the positive charge of the tail domain and as a result, reduces the affinity of NDC80 complexes for the negatively charged microtubule lattice. **(B)** Oligomerization. In this model, a dephosphorylated tail domain functions to oligomerize adjacent NDC80 complexes, which promotes high affinity NDC80-complex-microtubule binding. Upon phosphorylation of the tail domain, complex oligomerization is no longer favored, possibly due to a decrease in affinity of a phosphorylated tail domain for a negatively charged region within the CH domain of Hec1. **(C)** Co-factor recruitment. In this model, a dephosphorylated Hec1 tail domain recruits kinetochore-associated microtubule binding proteins or protein complexes to promote high affinity NDC80 complex-microtubule binding. In contrast, a phosphorylated tail domain restricts co-factor recruitment. As discussed in the text, these models are not mutually exclusive.

One mechanism by which the Hec1 tail domain may promote NDC80 complex binding to microtubules is one in which the tail directly contacts the microtubule lattice. The Hec1 tail domain is enriched in positively charged amino acids (isoelectric point ∼11), while the microtubule surface is enriched in negatively charged residues, many of which are within the unstructured C-terminal acidic tail domains of alpha and beta tubulin, which extend outward from the microtubule surface (Ponstingl et al., 1979; Sackett, 1995; Nogales et al., 1998, 1999; Löwe et al., 2001; Roll-Mecak, 2015). As such, electrostatic interactions may promote Hec1 tail-microtubule binding to provide an additional microtubule contact point within the NDC80 complex (Figure 3A). Consistent with this prediction, isolated tail domain fragments from human Hec1 directly bind microtubules *in vitro* (Miller et al., 2008; Alushin et al., 2012), and removal of the C-terminal tubulin tails (via limited protease digestion) leads to reduced affinity of NDC80 complexes for microtubules (Ciferri et al., 2008). Furthermore, Tooley et al. (2011) demonstrated that NDC80 complexes containing Hec1 tail domain mutants in which ten positively charged lysines and arginines were substituted with neutral alanines bound to microtubules with reduced affinity compared to wild-type complexes. Expression of these “neutral tail” Hec1 mutants also compromised kinetochore-microtubule attachments in cells (Tooley et al., 2011). Although these experiments support the notion that the Hec1 tail directly contacts the microtubule lattice, it is important to note that this domain is not sufficient for high-affinity NDC80-microtubule, or kinetochore-microtubule interactions. Notably, single point mutations in the Hec1 CH domain (within the “toe” domain) significantly reduce NDC80 complex-microtubule binding *in vitro*, and prevent formation of kinetochore-microtubule attachments in cells (Ciferri et al., 2008; Sundin et al., 2011; Tooley et al., 2011; Cheerambathur et al., 2013; Lampert et al., 2013). These defects in cells cannot be rescued by additional mutation of the Hec1 tail domain in which all Aurora B kinase target sites are mutated to prevent phosphorylation, which on its own results in hyper-stabilization of kinetochore-microtubule attachments (Sundin et al., 2011). An important question is why the NDC80 complex would require a second microtubule-binding site within the tail? One possibility is that in cells, a second microtubule binding domain would ensure that kinetochores remain bound to microtubules under conditions that might otherwise favor detachment. One such scenario might be poleward-moving kinetochores, where attached microtubules are predominantly depolymerizing, a state that may be unfavorable for microtubule binding by the Hec1 toe domain (Alushin et al., 2010; Schmidt et al., 2013).

The direct tail-microtubule binding model has also been useful for explaining how phosphorylation of the tail domain regulates kinetochore-microtubule attachment stability. In this model, addition of phosphate groups by Aurora kinases, or introduction of phospho-mimetic mutations in the Hec1 tail – both of which reduce the positive charge of the tail – are predicted to decrease NDC80 complex-microtubule binding affinity *in vitro* (Figure 3A). This is indeed the case (Cheeseman et al., 2006; Umbreit et al., 2012; Zaytsev et al., 2015). In fact, as mentioned above, NDC80 complexes with incrementally increasing numbers of phospho-mimetic substitutions in the Hec1 tail bind to microtubules with a corresponding step-wise decrease in affinity (Zaytsev et al., 2015). While data from numerous studies support a model in which phosphorylation of the Hec1 tail domain directly affects its interaction with microtubules, they do not rule out alternative NDC80 complex-intrinsic (i.e., in the absence of other factors) modes of regulating NDC80 complex-microtubule binding. For example, it is possible that the tail domain interacts directly with the Hec1 CH domain to influence CH domain-mediated microtubule binding. Given its contour length of ∼20 nm, this is indeed feasible. In such a model, phosphorylation of the tail may weaken kinetochore-microtubule attachments by enhancing the interaction between the Hec1 tail and CH domains, thereby preventing the CH domain from interacting with the microtubule lattice (Ciferri et al., 2008; Umbreit et al., 2012).

### Models for Attachment Stabilization and Regulation by NDC80 Complex Oligomerization

Multiple studies have suggested that the Hec1 tail domain promotes NDC80 complex-microtubule binding by affecting oligomerization of NDC80 complexes (Figure 3B; Alushin et al., 2010, 2012). It is well established that the NDC80 complex binds to microtubules in a cooperative manner (Ciferri et al., 2008; Alushin et al., 2010; Umbreit et al., 2012; Zaytsev et al., 2015; Helgeson et al., 2018), and that NDC80 complex oligomerization promotes high affinity interactions with microtubules (Powers et al., 2009; Volkov et al., 2018; Huis in’t Veld et al., 2019). A structural study from Alushin et al. (2010) suggested that this propensity to self-associate may be mediated by the Hec1 tail domain. In this study, the authors employed cryo-EM to obtain high-resolution electron density maps of NDC80^Bonsai^ complex-decorated microtubules that allowed for docking of the solved crystal structures of both tubulin and NDC80^Bonsai^ lacking the Hec1 tail domain. Electron densities were observed between adjacent NDC80 complexes that were not present in the crystal structures, and therefore the authors attributed these densities to the Hec1 tail (Alushin et al., 2010). Additionally, they reported that NDC80 complexes bound to microtubules in clusters of ∼6–8 complexes, and that deletion of the Hec1 tail reduced the number of complexes per cluster (Alushin et al., 2010). In a subsequent study, the authors found that the tail domain contains two functionally distinct zones: zone one (amino acids 41–80), which contributes to both NDC80 complex oligomerization and microtubule binding; and, zone two (amino acids 1–20), which contributes only to NDC80 complex oligomerization (Alushin et al., 2012). Hec1 tail phosphorylation has also been suggested to regulate NDC80 complex-microtubule binding affinity by modulating NDC80 complex oligomerization (Figure 3B). Specifically, Alushin et al. (2012) found that the number of microtubule-bound NDC80 complexes per cluster decreased when the complexes contained phospho-mimetic substitutions in the Hec1 tail domain (Alushin et al., 2012). As a consequence, the authors proposed that NDC80 complex-NDC80 complex interactions – which promote high microtubule-binding affinity – are facilitated by tail dephosphorylation.

Although these studies support the notion that the Hec1 tail domain facilitates high affinity microtubule binding through phosphoregulated oligomerization of NDC80 complexes, several lines of evidence indicate that this may not be the case. For instance, multiple studies have reported that phospho-mimetic substitutions in the Hec1 tail decrease microtubule-binding affinity of single NDC80 complexes independently of their oligomerization (Umbreit et al., 2012; Zaytsev et al., 2015). Furthermore, neither tail deletion nor phospho-mimetic mutants of Hec1 affect cooperative microtubule binding (Umbreit et al., 2012; Zaytsev et al., 2015), and tail-less human NDC80 complexes can still assemble into oligomers that bind microtubules with high affinity (Huis in’t Veld et al., 2019). Thus, while it remains possible that the Hec1 tail domain – and the phosphorylation state thereof – contributes to NDC80 complex oligomerization, it does not appear to be a critical effector for assembly or activity of NDC80 complex oligomers.

### Models for Attachment Stabilization and Regulation by Co-factor Recruitment

In a third model, the Hec1 tail may regulate kinetochore-microtubule attachment stability in cells by recruiting additional microtubule-binding proteins to the kinetochore (Figure 3C). During mitotic progression, several candidate factors localize to kinetochores coincident with Hec1 tail dephosphorylation and increased microtubule attachment stability. One of these is the Ska complex discussed above (Jeyaprakash et al., 2012; Cheerambathur et al., 2017). In metazoan cells, the Ska complex loads to kinetochores in an NDC80 complex-dependent manner, where it contributes to the establishment of stable kinetochore-microtubule attachments and is required for silencing the spindle assembly checkpoint (Hanisch et al., 2006; Daum et al., 2009; Gaiatanos et al., 2009; Guimaraes and DeLuca, 2009; Raaijmakers et al., 2009; Theis et al., 2009; Auckland et al., 2017). In a recent EM study using recombinant human proteins, it was found that NDC80^Bonsai^ complexes recruit “V”-shaped structures to the microtubules that were posited to be Ska complexes based on their size and shape (Jeyaprakash et al., 2012; Janczyk et al., 2017). Mutagenesis of the C-terminal half of the Hec1 tail to reduce its positive charge reduced clustering of the microtubule-bound NDC80 complexes, and also the incidence of the “V”-shaped structures on microtubules (Janczyk et al., 2017). The authors correlated this finding with human cell studies in which expression of this Hec1 mutant exhibited reduced kinetochore localization of the Ska complex. From this work, the authors concluded that the Hec1 tail plays a direct role in oligomerizing NDC80 complexes, and in recruiting the Ska complex to NDC80 complexes at the kinetochore-microtubule interface. These findings, however, contrast with a number of other studies that examined the Ska complex-NDC80 complex interaction. For example, several groups have reported that the tail domain of human Hec1 is dispensable for Ska complex-mediated enhancement of NDC80 complex-microtubule binding *in vitro* (Helgeson et al., 2018; Huis in’t Veld et al., 2019; Wimbish et al., 2019), and for kinetochore recruitment of Ska complexes in *C. elegans* and human cells (Cheerambathur et al., 2017; Wimbish et al., 2019). Instead, evidence suggests that the Ska complex contacts the NDC80 complex within the extended coiled-coil domain. For instance, multiple studies have reported that NDC80^Bonsai^ complexes, which are missing most of this internal coiled-coil, are unable to interact with Ska complexes (Zhang et al., 2017; Huis in’t Veld et al., 2019; Wimbish et al., 2019), presumably because this region mediates the interaction, a notion supported by cross-linking/mass spectrometry data (Helgeson et al., 2018). Thus, although NDC80 complex oligomerization may be part of the mechanism by which Ska complexes enhance NDC80 complex-microtubule binding, this is likely a Hec1 tail-independent phenomenon.

The phosphorylation state of the Hec1 tail has also been implicated in regulating recruitment of the Ska complex to kinetochores (Figure 3C). Expression of non-phosphorylatable Hec1/Ndc80 tail domain mutants in *C. elegans* embryos resulted in premature and enhanced recruitment of Ska complexes to kinetochores, as well as hyper-stabilized kinetochore-microtubule attachments (Cheerambathur et al., 2017). The authors found that these hyper-stable attachments could be rescued by depletion of Ska complexes, suggesting that dephosphorylation of the tail strengthens microtubule attachments in a Ska complex-dependent manner. Recent work from our lab showed that, as in worms, expression of a non-phosphorylatable Hec1 tail domain mutant (9A-Hec1) resulted in premature stabilization of kinetochore-microtubule attachments, and increased recruitment of the Ska complex to kinetochores (Wimbish et al., 2019). However, in contrast to the worm study, these hyper-stable attachments persisted even upon Ska complex depletion (Wimbish et al., 2019). Thus, the mechanism by which tail phosphorylation modulates kinetochore-microtubule attachments appears to differ somewhat among organisms.

As mitosis progresses, dephosphorylation of the Hec1 tail, stabilization of kinetochore-microtubule attachments, and Ska loading onto kinetochores are all largely coincident (Hanisch et al., 2006; Auckland et al., 2017). This leads to speculation that these events are functionally interdependent. Given the wealth of data demonstrating an NDC80 complex-intrinsic mechanism for phosphoregulation of microtubule binding affinity (i.e., additional co-factors are not required) (Cheeseman et al., 2006; Alushin et al., 2012; Umbreit et al., 2012; Zaytsev et al., 2015), it is almost certain that the mechanism for modulating kinetochore-microtubule attachment stability in cells involves, at least in part, direct regulation of NDC80 complex-microtubule binding affinity. Based on the available data, it is likely that the increased loading of the Ska complex to kinetochores is a consequence of increased microtubule occupancy upon Hec1 tail dephosphorylation, rather than a cause. It is possible, however, that the phosphorylation state of the Hec1 tail influences recruitment of other kinetochore-associated microtubule binding proteins aside from the Ska complex. These may include factors such as Astrin/SKAP, or Cdt1, both of which also load on to kinetochores as kinetochore-microtubule attachments are stabilized, and as mitosis progresses (Manning et al., 2010; Schmidt et al., 2010; Dunsch et al., 2011; Varma et al., 2012). Addressing these possibilities will be an important avenue of future investigation.

In summary, cells likely employ multiple mechanisms, including aspects of the three described above, to ensure precise regulation of kinetochore-microtubule attachments. An important goal for the future will be to determine how Hec1 tail domain phosphorylation functions in concert with other kinetochore-associated microtubule binding proteins to affect kinetochore-microtubule attachment dynamics and stability.

## Concluding Remarks

The small unstructured Hec1 tail domain clearly plays an important role at the kinetochore-microtubule interface during mitosis. Given the large body of work dissecting the role of phosphorylation of the Hec1 tail in regulating this interface, its role in coordinating temporal regulation of kinetochore function is becoming somewhat clear. Many questions still remain, however, including how the activities of the kinases and phosphatases that act on this domain are spatially and temporally regulated to ensure appropriate kinetochore-microtubule attachment strength.

In contrast to the well-defined role for the tail domain in regulation of kinetochore-microtubule attachments, its role in their establishment and maintenance is still not entirely resolved. Recent studies of human NDC80 complexes in cells and *in vitro* suggest that the tail domain is not explicitly required for generating kinetochore-microtubule attachments, but instead, plays a more nuanced role in harnessing the forces from dynamic microtubule plus-ends to power chromosome movement and silence the spindle assembly checkpoint. These studies, together with those carried out over the last decade in a number of eukaryotic species, suggest a more conserved role for the Hec1/Ndc80 tail domain across organisms.

## Author Contributions

JD and RW researched, wrote, and edited the manuscript.

## Conflict of Interest

The authors declare that the research was conducted in the absence of any commercial or financial relationships that could be construed as a potential conflict of interest.
